# *N*-[4-(2-Morpholino­eth­oxy)phen­yl]acetamide monohydrate

**DOI:** 10.1107/S1600536810053675

**Published:** 2011-01-08

**Authors:** Anuradha Gurumoorthy, Vasuki Gopalsamy, Ramamurthi. K, Poonam Piplani, Ruchi Malik

**Affiliations:** a Department of Physics, Saveetha School of Engineering, Saveetha University, Chennai-5, India; b Department of Physics, Kunthavai Naachiar Government Arts College (w) (Autonomous), Thanjavur-7, India; c Crystal Growth and Thin Film Laboratory, School of Physics, Bharathidasan University, Tiruchirappalli-24, India; d University Institute of Pharmaceutical Sciences, Panjab University, Chandigarh-14, India

## Abstract

In the title compound, C_14_H_20_N_2_O_3_·H_2_O, the geometry about the morpholine N atom implies *sp* ^3^ hybridization. In the crystal, symmetry-related mol­ecules are linked by inter­molecular N—H⋯O, O—H⋯O and O—H⋯N hydrogen bonds, forming infinite chains along the *b* axis. The chain structure is further stabilized by intra­molecular C—H⋯O inter­actions.

## Related literature

For related structures, see: Ahmad *et al.* (2009 ▶); Fun *et al.* (2010 ▶); Gowda *et al.* (2009*a* ▶,*b* ▶); Ma *et al.* (2009 ▶).
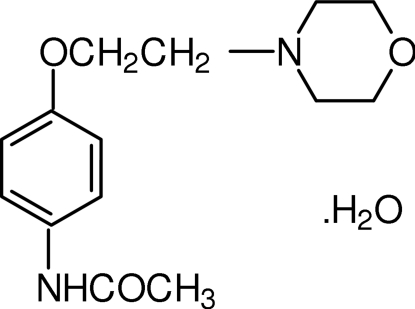


## Experimental

### 

#### Crystal data

C_14_H_20_N_2_O_3_·H_2_O
*M* *_r_* = 282.34
Triclinic, 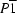

*a* = 7.0560 (3) Å
*b* = 10.2859 (6) Å
*c* = 10.7234 (6) Å
α = 87.572 (3)°
β = 73.326 (3)°
γ = 79.876 (3)°
*V* = 733.92 (7) Å^3^
*Z* = 2
Mo *K*α radiation
μ = 0.09 mm^−1^
*T* = 293 K
0.30 × 0.25 × 0.20 mm

#### Data collection

Bruker SMART CCD area-detector diffractometer
Absorption correction: multi-scan (*SADABS*; Bruker, 2001 ▶) *T*_min_ = 0.603, *T*_max_ = 0.705
17239 measured reflections
3723 independent reflections
2697 reflections with *I* > 2σ(*I*)
*R*_int_ = 0.028

#### Refinement

*R*[*F* ^2^ > 2σ(*F* ^2^)] = 0.044
*wR*(*F* ^2^) = 0.138
*S* = 1.05
3723 reflections
194 parameters
H atoms treated by a mixture of independent and constrained refinement
Δρ_max_ = 0.20 e Å^−3^
Δρ_min_ = −0.19 e Å^−3^

### 

Data collection: *SMART* (Bruker, 2001 ▶); cell refinement: *SAINT* (Bruker, 2001 ▶); data reduction: *SAINT*; program(s) used to solve structure: *SHELXS97* (Sheldrick, 2008 ▶); program(s) used to refine structure: *SHELXL97* (Sheldrick, 2008 ▶); molecular graphics: *PLATON* (Spek, 2009 ▶) and *ZORTEP* (Zsolnai, 1997 ▶); software used to prepare material for publication: *WinGX* (Farrugia, 1999 ▶).

## Supplementary Material

Crystal structure: contains datablocks I, global. DOI: 10.1107/S1600536810053675/jh2248sup1.cif

Structure factors: contains datablocks I. DOI: 10.1107/S1600536810053675/jh2248Isup2.hkl

Additional supplementary materials:  crystallographic information; 3D view; checkCIF report

## Figures and Tables

**Table 1:** Hydrogen-bond geometry (Å, °)

*D*—H⋯*A*	*D*—H	H⋯*A*	*D*⋯*A*	*D*—H⋯*A*
N1—H1⋯O6^i^	0.860 (18)	2.157 (18)	3.0148 (17)	174.8 (14)
O6—H6*A*⋯O5	0.82 (2)	2.06 (3)	2.8640 (18)	165 (2)
O6—H6*B*⋯N2	0.86 (3)	2.11 (2)	2.9586 (17)	171 (2)
C4—H4⋯O1	0.93 (3)	2.32	2.890 (2)	120

Symmetry code: (i) 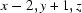.

**Fig. 1.**
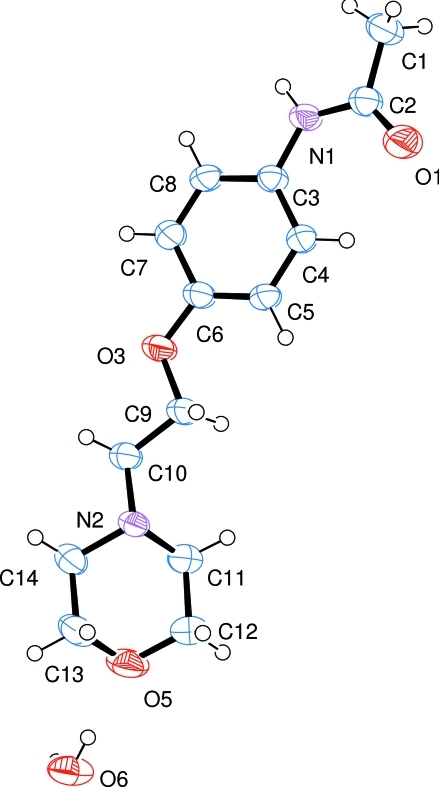
The molecular structure of the title compound.Displacement ellipsoids are drawn at the 50% probability level.

**Fig. 2.**
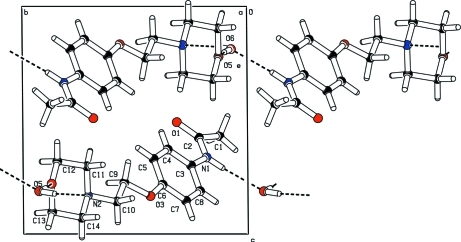
Crystal packing of the title compound with intermolecular N—H···O, O—H···O and O—H···N hydrogen bonds shown as dashed lines.

**Fig. 3.**
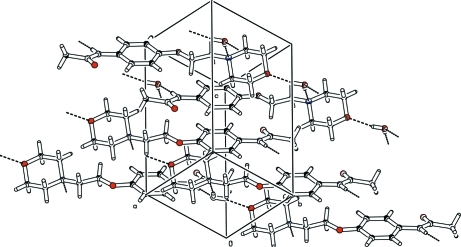
Crystal Packing of the title compound viewed along the b axis.

## supplementary crystallographic information

### Comment

Acetamide is important in the field of medicine as many biologically active compounds are synthesized by using acetamide. Benzanilides and benzamides exhibit wide range of biological activity and are extensively used in organic synthesis. Various N–substituted benzamides exhibit potent antiemetic activity (Ma *et al.*, 2009). As a part of studying the ring and side-chain substitutions on the crystal structures of chemically and biologically important class of compounds such as acetanilides, we report herein the crystal structure of the title compound(I). The conformation of the N—H bond in the structure of the title compound(I), is anti to the C═O bond and to the phenyl ring as it has been observed in the related structures containing acetamide derivatives (Gowda *et al.*,2009*a*; Gowda *et al.*,2009*b*; Ahmad *et al.*, 2009; Ma *et al.*, 2009; Fun *et al.*, 2010). Atom N1 has a trigonal configuration, the sum of three bond angles around them being 360°, whereas N2 atom is *sp*^3^ hybridized. The mean plane through the acetamide unit is inclined at a dihedral angle of 13.01 (11)° with respect to phenyl ring and 42.46 (8)° with respect to morpholine ring. The torsion angles and the least squares plane confirm that the morpholino ring is planar with the largest out-of-plane displacement of N2 (0.2458 (9) Å) and the phenyl ring is also planar with the root mean square deviation of 0.0034 Å.The morpholinoethoxy substitution at C6 [C4—C5—C6—O3=179.86 (13)°] is in anti-periplanar position. The exocyclic angle C2—N1—C3 [128.45 (11)°] deviates significantly from the normal value of 120°. This may be due to the intramolecular non-bonded interactions between atom O1 and H4 at C4 (O1······.H4 = 2.3159 Å). The widening of the exocyclic angle C5—C6—O3 [125.00 (12)°] deviate significantly from the normal value of 120° might be due to the consequence of repulsion between H5 and H9B at C9 (H5···H9B=2.3233 Å). The exocyclic angle O3—C9—C10 [103.45 (11)°] deviates by *ca* 6° from the tetrahedral value because of the intramolecular non-bonded interaction between O3 and H10A at C10 (O3···H10A = 2.3848 Å). The widening of the exocyclic angle C9—C10—N2[113.88 (11)°] from the normal value of 109° may be due to the repulsion between H9B and H11A [H9B···H11A = 2.3257 Å]. In the crystal structure, symmetry related molecules are linked by linear intermolecular N—H···O, O—H···O and O—H···N hydrogen bonds to form an infinite one-dimensional chain along the *b* axis.

### Experimental

*N*-[4-Hydroxyphenyl]acetamide (1.0 g, 6.62 mmol) was dissolved in ethyl methyl ketone (100 ml) and anhydrous potassium carbonate (3.0–4.0 g) was added. The reaction mixture was refluxed for 2 hrs. To it 4-(2-chloroethyl) morpholine hydrochloride (1.0 g, 6.68 mmol) was added and the reaction mixture was further refluxed with continuous stirring for 7 h. Reaction was monitored with the help of TLC. The slurry obtained was filtered, the solvent was removed under reduced pressure and the solid obtained was crystallized from a mixture of ethyl acetate and ether to afford the title compound(I) (1.43 g, 81.81%), mp 100–102°C.

### Refinement

Water H atoms were located in a difference Fourier maps and were included in the structure-factor calculations and isotropically refined. All the other H atoms were positioned geometrically and treated as riding on their parent atoms, with C—H = 0.93(aromatic),0.96(methyl) and 0.97Å (methylene),*N*—H = 0.86Å and refined using a riding model with *U*_iso_(H) = 1.2Ueq or 1.5Ueq (parent atom).In the absence of significant anomalous scattering effects,Friedel pairs were merged.

### Crystal data

**Table:**

C_14_H_20_N_2_O_3_·H_2_O	*V* = 733.92 (7) Å^3^
*M**_r_* = 282.34	*Z* = 2
Triclinic, *P*1	*F*(000) = 304
*a* = 7.0560 (3) Å	*D*_x_ = 1.278 Mg m^−^^3^
*b* = 10.2859 (6) Å	Mo *K*α radiation, λ = 0.71073 Å
*c* = 10.7234 (6) Å	µ = 0.09 mm^−^^1^
α = 87.572 (3)°	*T* = 293 K
β = 73.326 (3)°	Block, colourless
γ = 79.876 (3)°	0.30 × 0.25 × 0.20 mm

### Data collection

**Table:**

Bruker SMART CCD area-detector diffractometer	3723 independent reflections
Radiation source: fine-focus sealed tube	2697 reflections with *I* > 2σ(*I*)
graphite	*R*_int_ = 0.028
multi–scan	θ_max_ = 28.5°, θ_min_ = 2.0°
Absorption correction: multi-scan (*SADABS*; Bruker, 2001)	*h* = −6→9
*T*_min_ = 0.603, *T*_max_ = 0.705	*k* = −13→13
17239 measured reflections	*l* = −14→14

### Refinement

**Table:**

Refinement on *F*^2^	Primary atom site location: structure-invariant direct methods
Least-squares matrix: full	Secondary atom site location: difference Fourier map
*R*[*F*^2^ > 2σ(*F*^2^)] = 0.044	Hydrogen site location: inferred from neighbouring sites
*wR*(*F*^2^) = 0.138	H atoms treated by a mixture of independent and constrained refinement
*S* = 1.05	*w* = 1/[σ^2^(*F*_o_^2^) + (0.0701*P*)^2^ + 0.1005*P*] where *P* = (*F*_o_^2^ + 2*F*_c_^2^)/3
3723 reflections	(Δ/σ)_max_ < 0.001
194 parameters	Δρ_max_ = 0.20 e Å^−^^3^
0 restraints	Δρ_min_ = −0.19 e Å^−^^3^

### Special details

**Table:**

Geometry. All e.s.d.'s (except the e.s.d. in the dihedral angle between two l.s. planes) are estimated using the full covariance matrix. The cell e.s.d.'s are taken into account individually in the estimation of e.s.d.'s in distances, angles and torsion angles; correlations between e.s.d.'s in cell parameters are only used when they are defined by crystal symmetry. An approximate (isotropic) treatment of cell e.s.d.'s is used for estimating e.s.d.'s involving l.s. planes.
Refinement. Refinement of *F*^2^ against ALL reflections. The weighted *R*-factor *wR* and goodness of fit *S* are based on *F*^2^, conventional *R*-factors *R* are based on *F*, with *F* set to zero for negative *F*^2^. The threshold expression of *F*^2^ > σ(*F*^2^) is used only for calculating *R*-factors(gt) *etc*. and is not relevant to the choice of reflections for refinement. *R*-factors based on *F*^2^ are statistically about twice as large as those based on *F*, and *R*- factors based on ALL data will be even larger.

### Fractional atomic coordinates and isotropic or equivalent isotropic displacement parameters (Å^2^)

**Table:**

	*x*	*y*	*z*	*U*_iso_*/*U*_eq_	
C1	−1.0978 (2)	0.87498 (15)	0.43055 (17)	0.0553 (4)	
H1A	−1.1906	0.8271	0.4103	0.083*	
H1B	−1.0726	0.9451	0.3692	0.083*	
H1C	−1.1540	0.9115	0.5169	0.083*	
C2	−0.9045 (2)	0.78313 (14)	0.42318 (14)	0.0469 (3)	
C3	−0.54229 (18)	0.74747 (11)	0.30094 (12)	0.0375 (3)	
C4	−0.47022 (19)	0.64413 (12)	0.37122 (13)	0.0424 (3)	
H4	−0.5557	0.6167	0.4472	0.051*	
C5	−0.27177 (19)	0.58104 (12)	0.32949 (13)	0.0429 (3)	
H5	−0.2246	0.5123	0.3778	0.051*	
C6	−0.14488 (19)	0.62023 (13)	0.21659 (14)	0.0455 (3)	
C7	−0.2163 (2)	0.72379 (15)	0.14592 (15)	0.0541 (4)	
H7	−0.1311	0.7506	0.0696	0.065*	
C8	−0.4117 (2)	0.78676 (13)	0.18802 (14)	0.0465 (3)	
H8	−0.4575	0.8567	0.1403	0.056*	
C9	0.1304 (2)	0.45012 (14)	0.22585 (16)	0.0514 (4)	
H9A	0.0572	0.3790	0.2243	0.062*	
H9B	0.1241	0.4676	0.3152	0.062*	
C10	0.3450 (2)	0.41666 (14)	0.14270 (15)	0.0492 (3)	
H10A	0.3467	0.4195	0.0519	0.059*	
H10B	0.4183	0.4836	0.1564	0.059*	
C11	0.4840 (2)	0.28395 (14)	0.29775 (15)	0.0494 (3)	
H11A	0.3564	0.2987	0.3649	0.059*	
H11B	0.5564	0.3542	0.3040	0.059*	
C12	0.6040 (2)	0.15244 (16)	0.31893 (16)	0.0588 (4)	
H12A	0.6279	0.1526	0.4036	0.071*	
H12B	0.5276	0.0829	0.3180	0.071*	
C13	0.7573 (2)	0.12794 (15)	0.09625 (16)	0.0567 (4)	
H13A	0.6811	0.0594	0.0917	0.068*	
H13B	0.8851	0.1096	0.0296	0.068*	
C14	0.6448 (2)	0.25923 (15)	0.07117 (15)	0.0516 (4)	
H14A	0.7218	0.3279	0.0741	0.062*	
H14B	0.6257	0.2590	−0.0149	0.062*	
N1	−0.74111 (16)	0.81708 (11)	0.33615 (12)	0.0437 (3)	
N2	0.44861 (14)	0.28678 (10)	0.16958 (11)	0.0406 (3)	
O1	−0.89898 (17)	0.68586 (13)	0.49128 (14)	0.0801 (4)	
O3	0.05217 (15)	0.56571 (11)	0.16672 (11)	0.0655 (3)	
O5	0.79129 (15)	0.12632 (12)	0.22060 (12)	0.0651 (3)	
O6	1.21505 (19)	0.07084 (12)	0.18771 (14)	0.0651 (3)	
H1	−0.757 (2)	0.8870 (18)	0.2907 (16)	0.055 (4)*	
H6A	1.095 (4)	0.100 (2)	0.202 (2)	0.081 (6)*	
H6B	1.275 (4)	0.138 (2)	0.177 (2)	0.092 (7)*	

### Atomic displacement parameters (Å^2^)

**Table:**

	*U*^11^	*U*^22^	*U*^33^	*U*^12^	*U*^13^	*U*^23^
C1	0.0363 (7)	0.0512 (8)	0.0731 (10)	−0.0017 (6)	−0.0114 (6)	0.0060 (7)
C2	0.0388 (7)	0.0432 (7)	0.0555 (8)	−0.0029 (5)	−0.0119 (6)	0.0080 (6)
C3	0.0352 (6)	0.0306 (6)	0.0449 (7)	−0.0010 (5)	−0.0115 (5)	0.0012 (5)
C4	0.0393 (6)	0.0372 (6)	0.0455 (7)	−0.0012 (5)	−0.0082 (5)	0.0086 (5)
C5	0.0402 (6)	0.0348 (6)	0.0498 (7)	0.0009 (5)	−0.0123 (5)	0.0101 (5)
C6	0.0352 (6)	0.0399 (7)	0.0545 (8)	0.0027 (5)	−0.0085 (5)	0.0079 (6)
C7	0.0410 (7)	0.0529 (8)	0.0569 (9)	0.0004 (6)	−0.0037 (6)	0.0215 (7)
C8	0.0418 (7)	0.0398 (7)	0.0541 (8)	−0.0004 (5)	−0.0136 (6)	0.0156 (6)
C9	0.0386 (7)	0.0436 (7)	0.0610 (9)	0.0055 (5)	−0.0063 (6)	0.0141 (6)
C10	0.0376 (7)	0.0450 (7)	0.0563 (8)	0.0026 (5)	−0.0073 (6)	0.0121 (6)
C11	0.0454 (7)	0.0464 (7)	0.0543 (8)	0.0016 (6)	−0.0163 (6)	0.0011 (6)
C12	0.0510 (8)	0.0591 (9)	0.0628 (9)	0.0069 (7)	−0.0214 (7)	0.0086 (7)
C13	0.0365 (7)	0.0506 (8)	0.0714 (10)	0.0036 (6)	−0.0033 (6)	−0.0010 (7)
C14	0.0355 (7)	0.0531 (8)	0.0559 (8)	0.0011 (6)	−0.0027 (6)	0.0044 (6)
N1	0.0365 (5)	0.0343 (6)	0.0546 (7)	0.0021 (4)	−0.0099 (5)	0.0088 (5)
N2	0.0289 (5)	0.0398 (6)	0.0479 (6)	0.0017 (4)	−0.0076 (4)	0.0042 (4)
O1	0.0478 (6)	0.0771 (8)	0.0989 (10)	−0.0024 (6)	−0.0066 (6)	0.0474 (7)
O3	0.0381 (5)	0.0613 (7)	0.0755 (7)	0.0121 (5)	0.0012 (5)	0.0308 (6)
O5	0.0370 (5)	0.0686 (7)	0.0857 (8)	0.0073 (5)	−0.0218 (5)	0.0077 (6)
O6	0.0418 (6)	0.0487 (6)	0.1051 (10)	−0.0033 (5)	−0.0256 (6)	0.0132 (6)

### Geometric parameters (Å, °)

**Table:**

C1—C2	1.5007 (19)	C9—H9B	0.9700
C1—H1A	0.9600	C10—N2	1.4655 (16)
C1—H1B	0.9600	C10—H10A	0.9700
C1—H1C	0.9600	C10—H10B	0.9700
C2—O1	1.2151 (17)	C11—N2	1.4638 (18)
C2—N1	1.3483 (17)	C11—C12	1.510 (2)
C3—C4	1.3851 (17)	C11—H11A	0.9700
C3—C8	1.3883 (18)	C11—H11B	0.9700
C3—N1	1.4103 (15)	C12—O5	1.4250 (19)
C4—C5	1.3884 (17)	C12—H12A	0.9700
C4—H4	0.9300	C12—H12B	0.9700
C5—C6	1.3760 (19)	C13—O5	1.420 (2)
C5—H5	0.9300	C13—C14	1.497 (2)
C6—O3	1.3632 (15)	C13—H13A	0.9700
C6—C7	1.3873 (18)	C13—H13B	0.9700
C7—C8	1.3707 (18)	C14—N2	1.4695 (16)
C7—H7	0.9300	C14—H14A	0.9700
C8—H8	0.9300	C14—H14B	0.9700
C9—O3	1.4236 (16)	N1—H1	0.860 (18)
C9—C10	1.5079 (18)	O6—H6A	0.82 (2)
C9—H9A	0.9700	O6—H6B	0.86 (3)
			
C2—C1—H1A	109.5	N2—C10—H10B	108.8
C2—C1—H1B	109.5	C9—C10—H10B	108.8
H1A—C1—H1B	109.5	H10A—C10—H10B	107.7
C2—C1—H1C	109.5	N2—C11—C12	110.39 (12)
H1A—C1—H1C	109.5	N2—C11—H11A	109.6
H1B—C1—H1C	109.5	C12—C11—H11A	109.6
O1—C2—N1	123.54 (13)	N2—C11—H11B	109.6
O1—C2—C1	121.51 (13)	C12—C11—H11B	109.6
N1—C2—C1	114.95 (12)	H11A—C11—H11B	108.1
C4—C3—C8	118.57 (11)	O5—C12—C11	111.16 (13)
C4—C3—N1	124.49 (12)	O5—C12—H12A	109.4
C8—C3—N1	116.94 (11)	C11—C12—H12A	109.4
C3—C4—C5	120.70 (12)	O5—C12—H12B	109.4
C3—C4—H4	119.6	C11—C12—H12B	109.4
C5—C4—H4	119.6	H12A—C12—H12B	108.0
C6—C5—C4	120.00 (12)	O5—C13—C14	110.89 (13)
C6—C5—H5	120.0	O5—C13—H13A	109.5
C4—C5—H5	120.0	C14—C13—H13A	109.5
O3—C6—C5	125.00 (12)	O5—C13—H13B	109.5
O3—C6—C7	115.48 (12)	C14—C13—H13B	109.5
C5—C6—C7	119.51 (12)	H13A—C13—H13B	108.0
C8—C7—C6	120.36 (12)	N2—C14—C13	110.03 (11)
C8—C7—H7	119.8	N2—C14—H14A	109.7
C6—C7—H7	119.8	C13—C14—H14A	109.7
C7—C8—C3	120.84 (12)	N2—C14—H14B	109.7
C7—C8—H8	119.6	C13—C14—H14B	109.7
C3—C8—H8	119.6	H14A—C14—H14B	108.2
O3—C9—C10	103.45 (11)	C2—N1—C3	128.45 (11)
O3—C9—H9A	111.1	C2—N1—H1	118.1 (11)
C10—C9—H9A	111.1	C3—N1—H1	113.3 (11)
O3—C9—H9B	111.1	C11—N2—C10	111.50 (11)
C10—C9—H9B	111.1	C11—N2—C14	107.90 (11)
H9A—C9—H9B	109.0	C10—N2—C14	108.26 (10)
N2—C10—C9	113.88 (11)	C6—O3—C9	118.64 (10)
N2—C10—H10A	108.8	C13—O5—C12	109.68 (11)
C9—C10—H10A	108.8	H6A—O6—H6B	106 (2)
			
C8—C3—C4—C5	−0.1 (2)	C9—C10—N2—C11	−68.97 (16)
N1—C3—C4—C5	−179.93 (12)	C9—C10—N2—C14	172.48 (13)
C3—C4—C5—C6	−0.6 (2)	C13—C14—N2—C11	58.35 (15)
C4—C5—C6—O3	179.86 (13)	C13—C14—N2—C10	179.16 (13)
C4—C5—C6—C7	0.7 (2)	C5—C6—O3—C9	7.5 (2)
O3—C6—C7—C8	−179.30 (14)	C7—C6—O3—C9	−173.29 (14)
C5—C6—C7—C8	0.0 (2)	C10—C9—O3—C6	177.79 (13)
C6—C7—C8—C3	−0.7 (2)	C14—C13—O5—C12	59.49 (16)
C4—C3—C8—C7	0.8 (2)	C11—C12—O5—C13	−58.19 (17)
N1—C3—C8—C7	−179.40 (13)	O1—C2—N1—C3	4.2 (3)
O3—C9—C10—N2	−169.50 (12)	C1—C2—N1—C3	−175.68 (13)
N2—C11—C12—O5	58.19 (16)	C2—N1—C3—C4	−15.1 (2)
O5—C13—C14—N2	−60.57 (16)	C2—N1—C3—C8	165.11 (14)
O1—C2—N1—C3	4.2 (3)	C7—C6—O3—C9	−173.29 (14)
C1—C2—N1—C3	−175.68 (13)	C5—C6—O3—C9	7.5 (2)
C4—C3—N1—C2	−15.1 (2)	C6—O3—C9—C10	177.79 (13)
C8—C3—N1—C2	165.11 (14)	O3—C9—C10—N2	−169.50 (12)
C12—C11—N2—C10	−175.90 (11)	C9—C10—N2—C11	−68.97 (16)
C12—C11—N2—C14	−57.14 (15)	C9—C10—N2—C14	172.48 (13)

### Hydrogen-bond geometry (Å, °)

**Table:**

*D*—H···*A*	*D*—H	H···*A*	*D*···*A*	*D*—H···*A*
N1—H1···O6^i^	0.860 (18)	2.157 (18)	3.0148 (17)	174.8 (14)
O6—H6A···O5	0.82 (2)	2.06 (3)	2.8640 (18)	165 (2)
O6—H6B···N2^ii^	0.86 (3)	2.11 (2)	2.9586 (17)	171 (2)
C4—H4···O1	0.93	2.32	2.8896 (19)	120

Symmetry codes: (i) *x*−2, *y*+1, *z*; (ii) .
